# Non-invasive vestibular nerve stimulation (VeNS) reduces visceral adipose tissue: results of a randomised controlled trial

**DOI:** 10.1038/s41598-025-92744-9

**Published:** 2025-03-13

**Authors:** Erik Viirre, Julie Sittlington, David Wing, Ruth Price, Caomhan Logue, Daniel Moreno, Jeff Ledford-Mills, Cynthia Knott, Carel W. le Roux, David Grieve, Sinead Watson, Jason McKeown, Paul D. McGeoch

**Affiliations:** 1 Department of Neurosciences, University of California, San Diego, USA; 2 School of Biomedical Sciences, Ulster University, Coleraine, UK; 3 Exercise and Physical Activity Resource Center, University of California, San Diego, USA; 4 Altman Clinical and Translational Research Institute, University of California, San Diego, USA; 5 School of Medicine, University College Dublin, Dublin, Republic of Ireland; 6 School of Medicine, Dentistry and Biomedical Sciences, Queen’s University, Belfast, UK; 7 Neurovalens Ltd, Belfast, UK; 8 Center for Brain and Cognition, University of California, San Diego, USA; 9 Dept. of Neurosurgery, The Leland Stanford Junior University, Palo Alto, USA

**Keywords:** Visceral adipose tissue, Vestibular nerve stimulation, Vestibular, Obesity, Neuromodulation, Galvanic stimulation, Neuroscience, Medical research

## Abstract

Across multiple species, chronic vestibular stimulation activates hypothalamic regions involved in energy homeostasis and reduces body fat. This first-in-human randomised controlled trial evaluated the efficacy and safety of electrical vestibular nerve stimulation (VeNS) as a means of reducing excess body weight and fat. Overweight and obese adults were randomised 1:1 to receive 60 min of daily VeNS (n = 117) or sham stimulation (n = 124) for 6 months, together with a hypocaloric diet. The primary endpoints were weight loss based. Secondary endpoints included reduction in visceral adipose tissue (VAT). It is VAT, more than subcutaneous fat depots, which is particularly associated with the risks associated with obesity. The weight loss based primary endpoints were not met. However, mean change in VAT was significantly greater in the active (− 12.6%) versus the sham (− 4.7%) group (*p* = 0.03). This suggests that regular VeNS may cause a clinically meaningful reduction in VAT.

**Supplementary Information:**

The online version contains supplementary material available at 10.1038/s41598-025-92744-9.

## Introduction

It is projected that approximately half the global population will have developed the disease of obesity by 2035^1^. This is often attributed to “Western” diets high in simple sugars and saturated fats, coupled with ever more sedentary lifestyles^2^. Regardless of cause, excessive body fat, and in particular visceral adipose tissue (VAT), is associated with an elevated risk of both mortality and myriad morbidities^3–5^. Thus, there is compelling need for a safe effective intervention to reduce body fat.

Complex physiological mechanisms regulate energy intake and expenditure—a process known as energy homeostasis^6^. Although Claude Bernard recognised in the nineteenth century that the brain plays a role in this process, until recently its specific importance had been underappreciated^6^. A key neural structure involved is the arcuate nucleus (ARC) of the hypothalamus, which sits at the centre of the central melanocortin system (CMS). In the CMS two groups of neurons with antagonistic effects on appetite and energy expenditure fix a “set-point” for total body fat^2,7–9^. Normally, the CMS acts in response to various physiological parameters, such as circulating macronutrients, hormones, and neural inputs, to maintain body fat at or near this set-point. However, environmental factors, such as poor nutrition, stress and lack of sleep and exercise, are thought to affect the neurons of the CMS to push the body fat set-point up^2^. When this happens the CMS acts to maintain elevated fat levels, resulting in weight regain back to the body fat set-point after caloric restriction^2,7–9^.

**Fig. 1:**
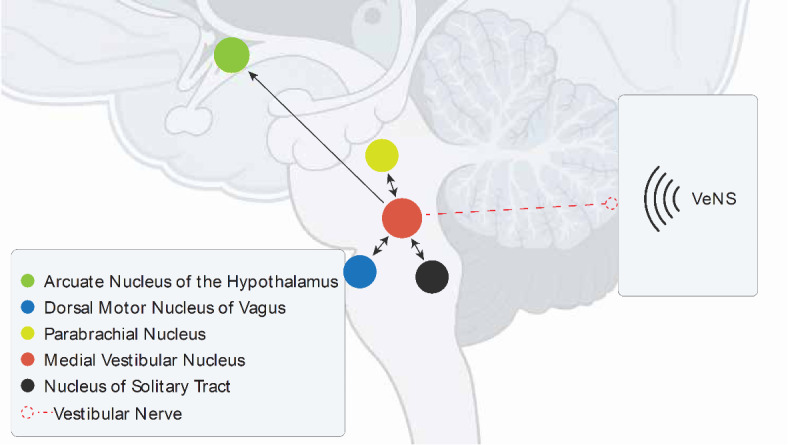
Vestibuloautonomic pathways in the brain stem and hypothalamus. Diagram of the non-invasive electrical vestibular nerve stimulation applied to the mastoid and subsequent projections through the medial vestibular nucleus to the vestibulohypothalamic and vestibuloautonomic pathways.

Most investigations of the neural inputs to the CMS have focused on parasympathetic projections from the nucleus of the solitary tract (NTS) to the ARC^9^. However, experiments on a range of mammals and birds have shown that long-term vestibular stimulation leads to a marked reduction in body fat, with either a slight increase or no change in muscle mass, and that this response is mediated by the otolith organs that detect horizontal and vertical movement^8,10,11^. This “metabolic shift” is thought to be mediated by a vestibulohypothalamic pathway, from the medial vestibular nucleus (MVe) to the ARC^8,11^. The MVe is the major projection target of the utricle, the otolith organ that detects horizontal movements^12^. The MVe contains anorexigenic CMS neurons and projects to similar neurons in the NTS, as well as having reciprocal connections to other brainstem homeostatic and gustatory sites, including the dorsal motor nucleus of the vagus and the parabrachial nucleus^7,8,13,14^ (Fig. 1). Teleologically, it is hypothesized that chronic vestibular stimulation is interpreted as a state of increased physical activity, during which time it is advantageous to liberate energy from fat stores^8^.

**Fig. 2:**
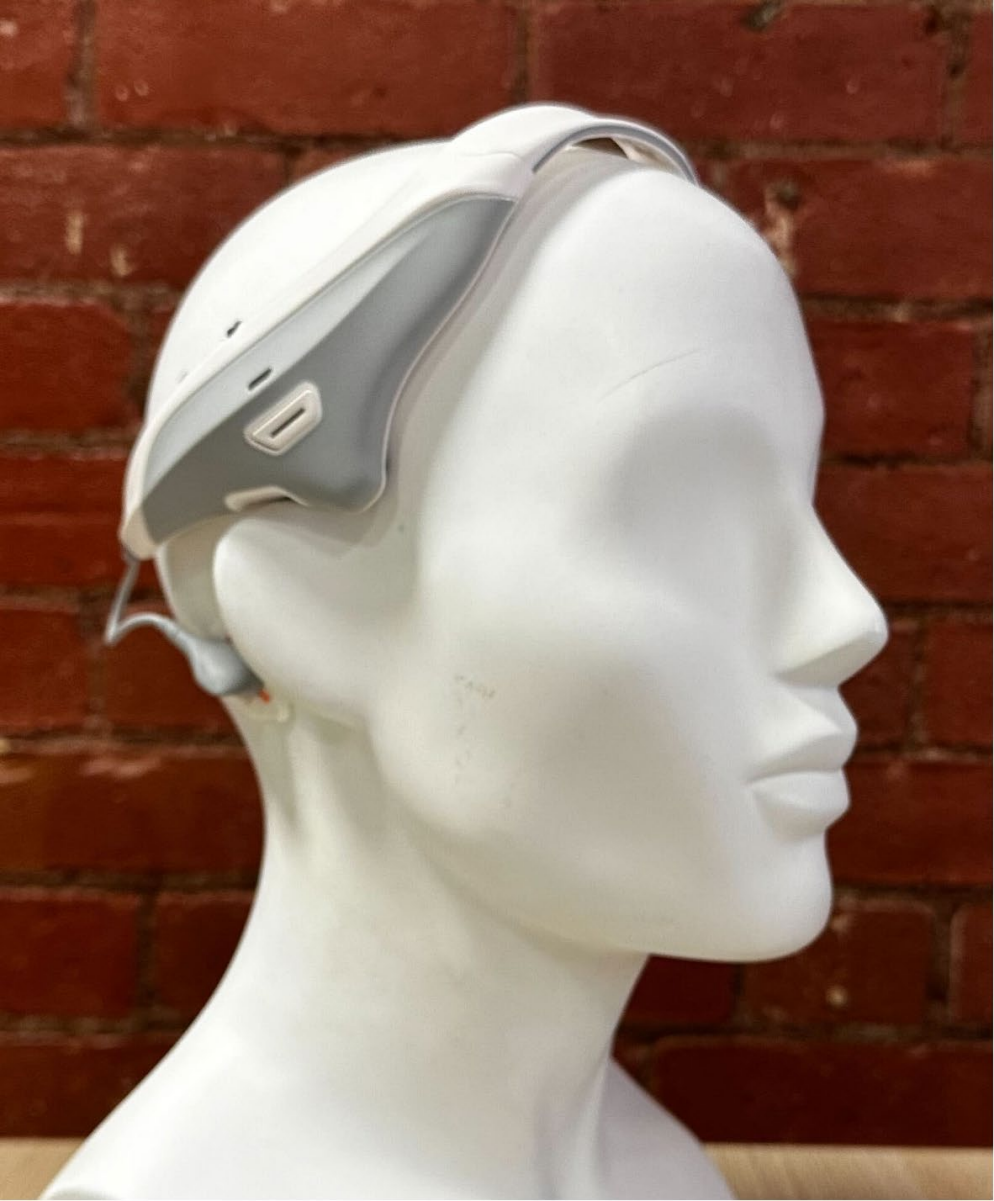
Modius Lean headset. *Note*: the external appearance of the sham device was identical.

Electrical Vestibular Nerve Stimulation (VeNS), also called galvanic vestibular stimulation, may be used to translate these findings to benefit human health, as at currents below 3 mA, VeNS preferentially activates the otolith organs^8,15^. Pilot data have also supported this premise^16^, whilst VeNS has been used in research for over a century and is reported to be safe^17^. Here, we present the results of a first-in-human multicentre randomised, sham-controlled, double-blind pivotal trial which was aimed at assessing the safety and efficacy of VeNS, in conjunction with a hypocaloric diet, as an innovative means of reducing excess weight and fat at 6 months (Fig. 2).

## Results

### Participants and treatment

Recruitment occurred between August 5, 2019, and October 15, 2021. A total of 317 participants were assessed for eligibility. Of these, 76 participants were deemed ineligible as they did not meet the eligibility criteria, and 241 were recruited. These 241 participants were randomly assigned to receive either the active (n = 117) or sham (n = 124) device and were included in the ITT analysis. (See Fig. 3a in supplementary information for trial profile). Retention rates did not differ between the active and control groups at 3 months (99/117, 85% vs. 98/124, 79%; *p* = 0.262) and at 6 months (91/117, 78% vs. 90/124, 73%; *p* = 0.351), respectively. Participants’ baseline characteristics were balanced between the active and sham device groups (Table 1).

**Fig. 3:**
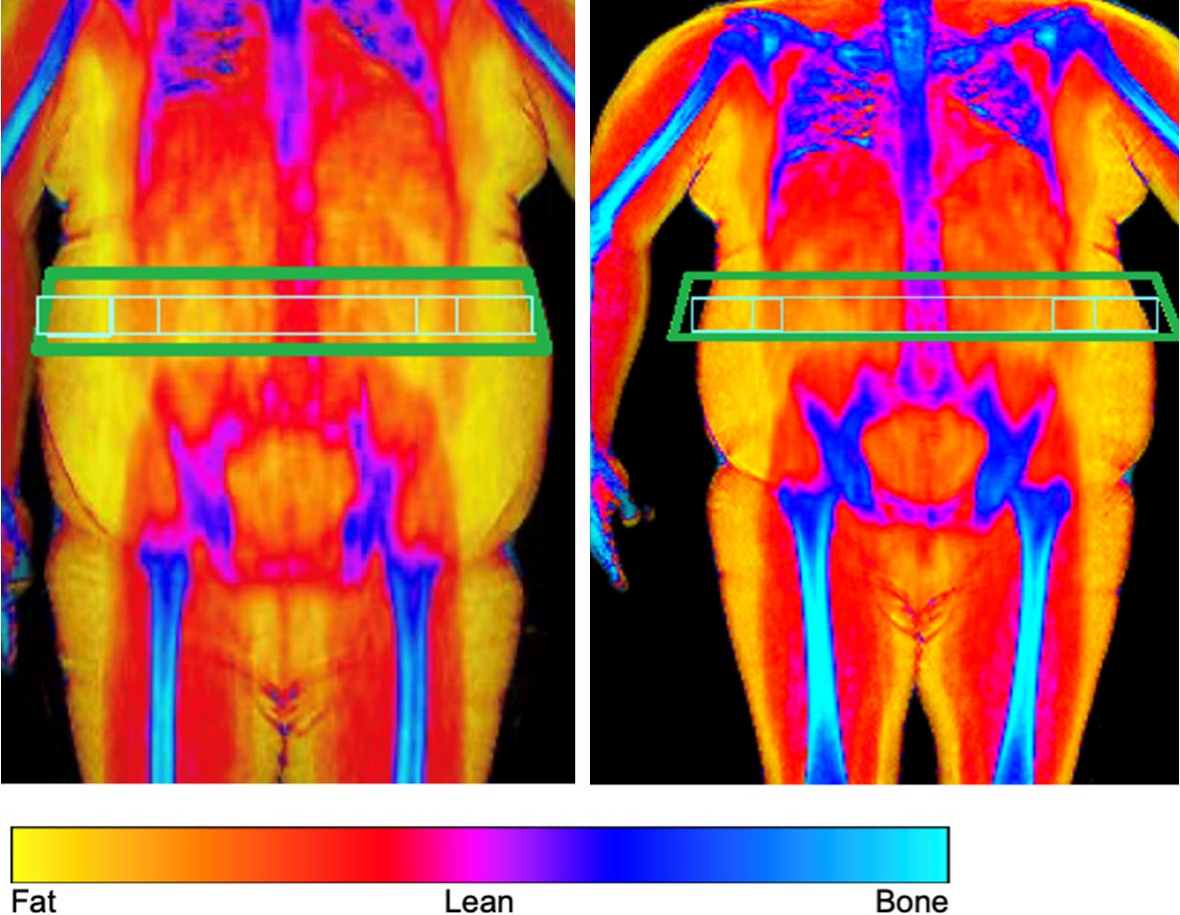
Example of a whole body DXA scan images at baseline (left) and 6 months (right). This male subject had an active device and reduced his total body fat by 23% and VAT by 44%. The Hologic Discovery DXA software automatically segmented body composition. The green boxes are placed for illustrative purposes and are the same area. They highlight the “android” box that the computer generates based on the height of the participant’s torso.

**Table 1:** Baseline characteristics according to randomisation group.

	Active device (n = 117)	Sham device (n = 124)	Overall (n = 241)
	Mean (SD)	Mean (SD)	Mean (SD)
Age (years)	45.5 (12.26)	47.2 (10.78)	46.3 (11.53)
Sex, n (%)			
Male	18 (15)	25 (20.2)	43 (18)
Female	99 (85)	99 (80)	198 (82)
Race, n (%)			
White	94 (80)	95 (77)	189 (78)
Black	6 (5)	9 (7)	15 (6)
Asian	5 (4)	6 (5)	11 (5)
Other	12 (10)	14 (11)	26 (11)
Weight (kg)	99.6 (20.07)	99.86 (20.37)	99.73 (20.18)
BMI (kg/m^2^)	36.94 (6.65)	35.81 (5.76)	36.36 (6.22)
Total Body Fat (kg)^a^	47.22 (13.84)	46.32 (12.20)	46.75 (13.00)
Visceral Adipose Tissue (kg)^b^	1.32 (0.69)	1.37 (0.74)	1.34 (0.72)

Data presented as mean (SD) or frequency (%).

^a^Total body fat: Active n = 116 versus Sham n = 124.

^b^Visceral adipose tissue: active n = 99 versus sham n = 113.

Compliance was assessed by the number of weeks participants used their allocated device for at least 5 h. Both groups had similar compliance rates (active group: 44% vs. sham group: 44%). The blinding analysis met the predefined criteria for adequate blinding of both the participants and trial coordinators across all the different timepoints, with the 95% CIs at or above 0.5 (see Table 5a in supplementary information for blinding results).

### Efficacy

At 6 months the ITT analysis found that the active group lost 2.91% of total body weight and the sham group lost 2.30%, the mean difference between the groups was not statistically significant [LSmeans [95% CIs]: −0.61% (−2.00, 0.79), *p* = 0.39] (Table 2). Categorically, the ITT analysis found that 21% (n = 25; 95% CIs: 0.14, 0.30) of the active group lost 5% or more of total body weight. Thus, neither of the primary endpoints achieved statistical significance and, as such, all the secondary endpoints were analysed from a hypothesis generating perspective.

**Table 2:** Mean weight loss from baseline to 6 months (ITT and per protocol primary outcome analysis).

	Active device n = 117	Sham device n = 124	Difference in LSmeans (95% CI)	*p*-value
	LSmean	LSmean		
% change in weight				
ITT analysis	−2.91	−2.30	−0.61 (−2.00, 0.79)	0.394
Per-protocol analysis*	−3.16	−2.32	−0.84 (−2.32, 0.64)	0.266

*Per protocol population (Active Device = 83 and Sham Device = 87) excludes participants who did not use the device for an average of 3.5 h a week for at least 4 of the first 6 months. Linear regression model adjusted for baseline weight and gender. LSmeans, Least square means; ITT, Intention to Treat analysis.

Secondary endpoints (Table 3) showed that the active group, compared with the sham group, achieved a higher reduction in VAT (ITT: −12.63% vs. −4.67%, *p* = 0.03) at 6 months (see Fig. 3 for examples of DXA scans). None of the other secondary outcomes differed statistically (Tables 3 and 4). Summaries of the exploratory secondary outcomes are presented in Supplementary Information (Additional Results, Table 2a–4a). A descriptive comparison analysis shows that the exploratory secondary outcomes were comparable between the two groups. The post-hoc analyses found that 44% of the active group and 30% of the sham group lost ≥ 5% total body fat (ITT: 51/116 vs. 37/124, *p* = 0.03), and 49% of the active group and 35% of the sham group lost ≥ 10% VAT (ITT: 49/99 vs. 39/113, *p* = 0.04).

**Table 3:** Key secondary outcomes (body composition) from baseline to 6 months according to randomisation group.

Secondary outcomes	Active device	Sham device	Difference in LSmeans (95% CI)	*p*
	LSmean change	LSmean change		
Visceral adipose tissue				
ITT analysis (A = 99, S = 113)	−12.63	−4.67	−7.96 (−15.26, −0.65)	0.033
PP analysis (A = 49, S = 53)	−12.29	−5.65	−6.63 (−11.57, −1.70)	0.009
Total body fat				
ITT analysis (A = 116, S = 124)	−7.07	−4.62	−2.45 (−5.91, 1.01)	0.165
PP analysis (A = 60, S = 58)	−6.77	−4.70	−2.08 (−5.21, 1.06)	0.192
Lean muscle mass				
PP analysis (A = 60, S = 58)	−0.36	0.05	−0.40 (−1.81, 1.00)	0.571
Trunk fat				
PP analysis (A = 60, S = 58)	−7.33	−5.73	−1.60 (−5.29, 2.08)	0.391

Data are presented as LS mean percentage change. Per protocol population includes participants with complete data and excludes any participants with major protocol deviations or participants who did not use the device for an average of three and a half hours a week for at least 4 of the first 6 months. Post-hoc analysis included ITT analysis for VAT and total body fat only. ITT—participants with missing baseline values were excluded. Linear regression model adjusted for baseline values (outcome) and gender. A, active device; S, sham device; LSmeans, Least square means; ITT, Intention to treat analysis; PP, Per-protocol analysis.

**Table 4:** Secondary outcomes from baseline to 6 months according to randomisation group.

Secondary outcomes	Active device	Sham device	Difference in LSmeans (95% CI)	*p*
	LSmean change	LSmean change		
Low density lipoprotein (A = 55, S = 55)	1.48	1.20	0.29 (−6.17, 6.74)	0.930
Atherogenic index (A = 55, S = 55)	−0.14	−0.32	0.18 (−0.03, 0.38)	0.087
C-reactive protein (A = 57, S = 56)	−0.01	−0.67	0.65 (−0.54, 1.85)	0.279
Bone mineral density^a^ (A = 60, S = 58)	0.28	0.06	−0.22 (−0.60, 1.03)	0.598
Total energy intake (A = 82, S = 85)	−164.60	−132.62	−31.98 (−279.03, 215.07)	0.799
IWQoL (A = 88, S = 89)	8.85	10.29	−1.44 (−5.05, 2.16)	0.430

Data are presented as LS mean absolute change and LS mean percentage change^a^. Analyses based on per protocol population which includes participants with complete data and excludes any participants with major protocol deviations or participants who did not use the device for an average of three and a half hours a week for at least 4 of the first 6 months. Linear regression model adjusted for baseline values (outcome) and gender.

A, active device; S, sham device; LSmeans, Least square means.

### Safety

A total of 108/238 (45%) participants in the safety analysis set reported having an AE during the 6-month study. The most common AEs reported were dizziness, fatigue, headache, ear pain, mild tinnitus, nausea, and skin irritation. There was no statistical difference between the active group and sham control group in the number of causally related AEs (active group 49/116 (42%) vs. sham group 48/122 (39%); *p* = 0.69). There were no SAEs reported during the study. The otoscope examination and audiogram test results indicated that the ear canal and hearing were not adversely impacted.

### Covid-19

A total of 178 participants (active n = 90 and sham n = 88) completed the Covid-19 questionnaire via the Modius app. Most participants reported that their appetite levels had increased (active n = 49 (54%) vs. sham n = 53 (60%)), and their physical activity levels had decreased (active n = 58 (64%) vs. sham n = 54 (61%) during this period (Supplementary Information, Table 5a).

## Discussion

It is well known that higher levels of adiposity are associated with an increased risk of morbidity and all-cause mortality^3–5,18^. Thus, although the primary endpoints relating to weight loss were not met, it is noteworthy that the active VeNS group showed an 8% greater reduction in VAT than the control group, as indicated by ITT analysis. This is because VAT is more metabolically active and pro-inflammatory than subcutaneous adipose tissue (SAT), so is more closely correlated with the adverse metabolic consequences of obesity, including cardiovascular disease, type 2 diabetes, and non-alcoholic fatty liver disease^19,20^. Moreover, reducing VAT generates a multifaceted improvement in cardiometabolic risk profile^5,19,20^. Although reductions in VAT do not always correlate with substantial weight loss, they are associated with a lower cardiovascular disease risk^21^. In fact, the strong correlation between VAT and metabolic risk profile is maintained even when controlling for weight and BMI^22^. Moreover, weight loss can occur with quite variable degrees of associated fat loss^23^, indicating the clinical importance of not simply considering weight loss in isolation.

An important limitation is that although reducing VAT is key to normalising physiology and reducing risk profile in obesity, weight loss measures were defined as the primary endpoints^5,20,21^. In fact, as originally designed a DXA measure of adiposity was to be the study’s primary endpoint, with weight loss as a secondary endpoint. The underlying rationale was that the animal literature suggested vestibular stimulation acted, via the hypothalamus, to specifically reduce fat storage^10,11,13^. However, after seeking regulatory input on the trial’s design, this was changed to the current endpoints on the basis that weight loss was the primary endpoint used in most other obesity trials. This reflects a controversy in the field that hopefully future studies will rectify, namely that despite the near ubiquitous focus of obesity and overweight studies on crude weight loss, the reduction of VAT is more clinically relevant.

Historically, part of the reason clinical trials have focused on weight loss is that it is practically much easier to measure weight than VAT. However, a particular strength of the present study is that incorporation of DXA analysis enabled additional accurate quantification of body composition including VAT^24^. The question as what constitutes a clinically meaningful reduction in VAT is still an open question^25^. Notably, the reduction seen in the active VeNS group is comparable to the value reported in a previous study with associated health benefits^26^. Recently, four cut-offs for VAT mass have been suggested, above which cardio-metabolic risk increases. These cut-offs, which were determined using DXA, are: 700 g in women and 1000 g in men under 40 years; and 800 g in women and 1200 g in men over 40 years^25^. These VAT cut-offs were proposed after this trial started; however, future trials could assess the effect of VeNS on individuals in these higher risk cohorts.

As the trial was focussed on assessing efficacy and safety of VeNS, we did not include follow-up post intervention. We are therefore unable to conclude whether the observed reductions in VAT and fat would be maintained long-term and after cessation of VeNS. Similarly, it is possible that conducting the study over a longer period than 6 months may have revealed greater weight loss. Another point to note is that the trial employed a standardized hypocaloric deficit for all participants, which historically has been a common practice in weight loss trials. However, possibly an individualized dietary program tailored to participants’ specific metabolic needs might have proven more effective, although conversely this could have made it difficult to analyse the isolated benefit of the Vestal Device. It is important to appreciate that the trial was mostly conducted during the height of the Covid-19 pandemic with various adverse consequences. This certainly contributed to the high dropout rate of 25% and led to missing data. As reflected in the Covid-19 questionnaires, the pandemic was an intensely stressful period that led to widespread weight gain throughout the UK and USA, which could be a potentially confounding factor^27^. Nonetheless, the fact that our trial was able to continue during the pandemic, with necessary modifications, and despite the extreme stresses of this period, demonstrated significant reductions in VAT and fat, represent a major strength. Another notable strength is the verisimilitude of the sham device, as shown by the blinding analysis.

Our observation that VeNS markedly reduced VAT and fat may be particularly important given that the underlying mechanism of action involves brainstem and hypothalamic homeostatic sites, with the putative purpose of gauging physical activity and determining when to liberate energy from fat depots. Compared to SAT, VAT is more metabolically active and plays a disproportionate role in energy metabolism. Reflecting this, VAT is highly vascularized and innervated, making it more responsive than SAT to the hormonal and neural cues that regulate fat mobilization^19,20^. We propose that the vestibulohypothalamic pathway activated by VeNS likely targets these physiological properties of VAT and its distinct role in metabolic regulation, promoting the selective mobilization of VAT for energy utilization while sparing other fat depots. This specificity may be explained by the hypothesized evolutionary role of vestibular inputs in signaling increased physical activity, during which energy demands are best met by quickly accessible fat stores such as VAT^8^. The reduction in VAT without concomitant muscle loss or significant changes in total body fat highlights the potential of VeNS to drive a beneficial redistribution of body composition, focusing on the fat depot most closely linked to adverse metabolic outcomes^19,20^.

The observed effect on VAT in the active group is comparable to that reported with glucagon-like peptide-1 (GLP-1) receptor agonists, which are becoming widely used for weight loss with dramatic effect^28–30^. Indeed, the reported VAT reduction at 36 weeks for liraglutide is 12.49%, albeit measured by MRI^30^. Of course, the observed VAT reduction in the active group must be tempered against the lack of improvement in other physiological parameters and requires replication. Nonetheless, this novel finding is clearly exciting and if replicated holds evident therapeutic value.

Moreover, about a third to a half of the weight loss produced by current treatments for obesity, such as hypocaloric diets is actually due to lean muscle loss^28,29^. This figure may even be higher with GLP-1 agonists, which are thought to act by increasing satiety^29^. Indeed, estimates for liraglutide range from 30 to 47% of total weight loss being lean muscle mass^29^, and in the STEP1 trial for semaglutide at 68 weeks, 38.6% of the weight loss in the active group was lean mass (n = 95).

The finding that VeNS, in conjunction with a weight management program, specifically reduces fat and VAT, is in keeping with the observation that animals subjected to chronic vestibular stimulation show a metabolic shift to a leaner physique^10,11^. Although GLP-1 agonists were originally thought to act via the gut, it is now thought that their primary mode of action is on the brain. If non-invasive targeting of metabolic pathways using VeNS can produce significant VAT loss, without associated significant muscle loss, then this would be of direct clinical relevance. Indeed, it would raise the question as to whether VeNS, when used together with a GLP-1 agonists, could have a synergistic effect to both increase VAT loss and ameliorate muscle loss. This is a clear direction for future research.

## Methods

### Study design

The trial was conducted between August 2019 and April 2022 at four sites—three in the US and one in the UK. These were: the University of California, San Diego (UCSD) where there were two sites—the Altman Clinical and Translational Research Institute (ACTRI) and the Exercise and Physical Activity Resource Center (EPARC); Texas Diabetes & Endocrinology (TDE) in Austin; and Ulster University (UU). The protocol was approved by: UCSD Institutional Review Board (IRB) (Ref: 181094) for ACTRI and EPARC; Advarra IRB (Ref: CR00383819) for TDE; and Northern Ireland Research Ethics Committee (Ref: 18/NI/0176) for UU. All methods were performed in accordance with the relevant guidelines and regulations. The study protocol is available here: https://clinicaltrials.gov/study/NCT03640286. The trial was registered with ClinicalTrials.gov under identifier NCT03640286 (registered: 21/08/2018). See the Supplementary Information for the Consolidated Standards of Reporting Trials (CONSORT) checklist and for additional information on the methods and results sections.

### Participants

The study recruited adults aged 22 to 80 years, with a body mass index (BMI) ≥ 27 kg/m^2^. Key exclusion criteria included a history of vestibular dysfunction, diabetes mellitus and pregnancy. Participants self-identified their gender as male or female. Supplementary Information (Participants) details all eligibility criteria. All participants gave written informed consent.

### Randomisation and masking

Participants were randomly assigned to receive either an active VeNS or a sham device, and all also received a lifestyle modification program. Random assignment to active or sham device was carried out at baseline via a block allocation procedure (block size = 2) in which two participants at a time were randomised 1:1. Stratification was performed by gender and centre. Sealed opaque envelopes containing allocated study device serial numbers were used to conduct this process. To ensure blinding of study staff these were prepared off-site by Compliance Solutions (CS), a third-party medical device regulatory consultancy (https://cslifesciences.com). The envelopes were sent by CS directly to the study sites. At the study sites eligible participants were enrolled and randomised by study coordinators. All participants and study staff (including those giving interventions, assessing outcomes, and analysing the data) were kept blinded. To ensure an adequate degree of verisimilitude, active sham devices were used which delivered an ineffective electrical tingling sensation to the skin with the aim of providing a convincing experience to those naïve to VeNS. Externally the investigational and sham devices appear identical.

### Procedures

At baseline, participants were provided with their allocated device, charging cable, an iPod pre-installed with the Modius app, alcohol wipes and electrodes. Study coordinators demonstrated how to effectively apply and operate their device (Supplementary Information: Procedures). All participants were instructed to use their allocated device for 60 min per day, at least five times a week for 6 months (See Fig. 2 for an image of the Modius Lean VeNS device). All participants were also prescribed a 600 kcal deficit hypocaloric diet and received counselling from a research dietitian at baseline and 3 months (Supplementary Information: Lifestyle Modification Program). Participants were additionally provided with weekly dietary support by a company called Clinical Trial Mentors (CTM) (https://ctmentors.com/).

When turned on, the active device (ML1000) started at 0 mA and the current could be increased in 0.1 mA increments up to a maximum of 1 mA. The waveform it delivered was a bipolar rectangular shape with a 50% duty cycle (Supplementary Information, Fig. 1a). The device was powered via a 3.75 V battery. The sham device delivered an ineffective stimulation for a limited period (30 s), before tapering down to zero over 20 s, conveying a sense of verisimilitude to naive users (Supplementary Information, Sham Device Design). Participants were instructed to quickly turn their allocated device up to the maximum comfortable level and stay at this for the duration. Usage of the device was restricted to 60 min per day by means of a software lock-out.

Baseline assessments were conducted face-to-face at each research site and participants were scheduled to return at 3 months (± 2 weeks) for a mid-trial assessment and at 6 months for the end of trial assessment. Supplementary Information (Table 1a) details the full schedule of study assessments. The key assessments were weight (measured wearing a gown on calibrated scales with an empty bladder) and automatically segmented measures of body composition derived from dual-energy x-ray absorptiometry (DXA) scanning. Due to the onset of the Covid-19 pandemic in March 2020, many of the 3 and 6-month assessments had to be undertaken remotely. In this case, participants were sent calibrated SECA medical grade scales and detailed instructions on how to weigh themselves. They recorded their weight by video and photograph and emailed these to the site. As such, some of the secondary outcome data could not be collected.

### Outcomes

The efficacy of the active device was quantified by two primary endpoints at 6 months: the mean percentage weight loss in comparison to the sham device and the proportion of participants who lose at least 5% body weight. The success criterion for this latter categorical endpoint of at least 5% of body weight was defined as the lower bound of the 95% confidence interval of the response rate in the active device group exceeding 50%. If the primary endpoints met the threshold for statistical significance, then the following secondary endpoints, assessed at baseline and 6 months, would be statistically analysed hierarchically to make labelling claims: percentage visceral adipose tissue (VAT) loss (assessed by whole body DXA); percentage change in low density lipoprotein (LDL) from lipid profile; percentage fat loss (assessed by whole body DXA); percentage change in lean muscle mass (assessed by whole body DXA); change in atherogenic index (ratio of total cholesterol to HDL from lipid profile); change in systemic inflammation (high-sensitivity C-reactive protein); change in total energy intake (in kcal—assessed using two-day 24-h dietary recall); and change in Duke Impact of Weight on Quality of Life (IWQoL) questionnaire total score.

The following exploratory secondary endpoints were also assessed at baseline and at 6 months (presented as descriptive summaries in Supplementary Information, Table 4a): percentage trunk fat loss and change in bone mineral content (by whole-body DXA); fasting glucose; glycosylated haemoglobin (HbA1c); physiological parameters—blood pressure, heart rate, waist and hip circumference (at USA sites only); body mass index (BMI); and medication use changes, including cardiovascular medication alterations and their potential impact on weight status (Supplementary Information: Outcomes).

The following were also assessed: device usage data as uploaded by the Modius app, which were used to assess compliance; data on average current intensity and electrical impedance (also uploaded by the app and kept inaccessible from participants and study staff to avoid unblinding); mentor support usage data (hours per week); and the impact of the Covid-19 pandemic on participants’ diet and physical activity, which was assessed using a questionnaire distributed via the Modius app. The effectiveness of participant blinding was assessed at baseline (after using the device for one session), after 1 month of use and after 6 months of usage. Participants were asked to indicate if they believed they received the active device, sham device or if they “did not know”. The effectiveness of study coordinator blinding was similarly assessed at 6 months.

Adverse events (AE) were actively assessed using a questionnaire that the participants completed at 1, 3 and 6 months, or if they complained of an AE (Supplementary Information, Fig. 2a). Participants were also provided with study contact details and encouraged to report any adverse events or concerns. The following were used to assess safety: frequency of AEs and Adverse Device Events (ADE); frequency of serious adverse events (SAE) and serious adverse device events (SADE); adverse event monitoring questionnaire; and outcome of ear canal and hearing assessments (Supplementary Information: Audiology Assessments).

### Statistical analysis

The statistical analysis plan (SAP) was developed by Exploristics, which is a biostatistics and trial design company (https://exploristics.com). The statistical analysis was undertaken by GEM Programming Solutions, which is a statistical CRO (https://gemprogramming.com). Exploristics carried out the power calculation by means of multiple simulations (each with 1000 iterations) to determine an appropriate sample size. Simulation assumptions were informed by our pilot data (Supplementary Information: Power Calculation)^16^. Under these assumptions, with a sample size of 180 participants randomised 1:1 active: sham control, there was a greater than 80% power to demonstrate both the weight loss mean and categorical acceptance criteria at the 6-month time point. Anticipating a drop-out rate of about 10%, the aim was to recruit 200 participants in the study across all sites. Note, due to the high dropout rate this was allowed to increase to 241.

All data collected for the study were analysed using SAS® v 9.4. The threshold of significance was set at the 5% alpha level, (i.e., *p* < 0.05; two-sided). The primary analyses were performed on the Intention to Treat (ITT) population, which included all randomised participants who had baseline data available. A multiple imputation method was used to deal with missing data and losses to follow-up. The missing data were imputed multiple times to generate 75 imputations per missing value, the variability between imputed values represents the uncertainty over the missing value. For the primary analysis, imputed values were predicted total body weight loss values obtained from a linear regression model with observed total body weight loss data as the dependent endpoint, and randomisation group, gender, and baseline weight used as covariates. The results from the 75 complete datasets were then combined to produce an estimate for the primary endpoint, which reflects the uncertainty due to the missing values. An ITT population was also used for the analysis of VAT and total body fat (secondary outcomes) with missing data similarly imputed.

A per-protocol (PP) analysis was undertaken on participants who used the device for at least an average of 3.5 h a week for at least four of the first six months. The safety analyses were performed on all randomised participants who received either the active or sham devices and recorded at least one session of use, defined as usage on at least one day. For the continuous efficacy (primary) endpoint, mean percentage weight loss at 6 months, the difference between randomisation groups was assessed using a linear model controlling for gender and baseline weight. A Fisher’s exact test was used to statistically compare the response rates (the number who achieved ≥ 5% weight loss) in each randomisation group. Criteria for declaring success based on the primary efficacy criteria were predefined (Supplementary Information, Primary Efficacy Criteria).

All secondary endpoints were subjected to statistical analysis; however, if the primary endpoints were not met, no formal statements of statistical significance can be declared in relation to the labelling claim associated secondary endpoints. These secondary endpoints assessed the difference between randomisation groups by considering the change from baseline to the 6-month time point using a linear model adjusting for the baseline value and gender. The first of these secondary endpoints was VAT. The statistical hypotheses for VAT and the other labelling claim associated secondary endpoints are detailed in Supplementary Information (Secondary Outcomes). The exploratory secondary endpoints were assessed through summary and descriptive statistics. Assessments of safety and the impact of the pandemic on subjects were evaluated by descriptive summaries only.

Due to the Covid-19 pandemic 31 participants in the active and 32 in the sham group were unable to attend for DXA scans at 6 months (Supplementary Information, Outcomes). However, 18 of the UCSD participants had inadvertently had off-schedule 3-month DXA scans prior to the onset of the pandemic. After, informing and seeking approval from the UCSD IRB, data from these scans were included in the missing data analysis. Following completion of the analyses described in the SAP, key DXA secondary outcomes prompted further investigation with the following two post-hoc analyses. First, the percentage of participants at 6 months achieving total body fat loss ≥ 5%. Second, the percentage VAT loss of ≥ 10%. Fisher’s exact test was used in both these analyses to compare the response rate in each group.

The approach to the blinding analysis is described in Supplementary Information (Blinding Assessment). If the lower bound of the calculated 95% asymptotic confidence intervals is above 0.5, then a suitable level of blindness has been achieved. Due to the anticipated good safety profile of the device, a data monitoring committee was not necessary.

## Electronic supplementary material

Below is the link to the electronic supplementary material.

Supplementary Material 1

## Data Availability

Data sharing requests relating to data in this paper will be considered after the publication acceptance date and after this product and indication has been approved in both the United States and European Union. Access will be provided after obtaining approvals through contact with the corresponding author and after receipt of a signed data sharing agreement. Data sharing requests will be considered for research or academic purposes only. The study protocol and statistical analysis plan are available here: https://clinicaltrials.gov/study/NCT03640286.
