# Supplementary material for: Non-invasive vestibular nerve stimulation (VeNS) reduces visceral adipose tissue: results of a randomised controlled trial
Source: Sci Rep. 2025 Mar 13;15:8753. doi: 10.1038/s41598-025-92744-9 (PMC11906803; doi:10.1038/s41598-025-92744-9)
Supplement: Supplementary file 1 — Supplementary Material 1 [file 41598_2025_92744_MOESM1_ESM.docx]

**SUPPLEMENTARY INFORMATION**

**ADDITIONAL STUDY METHODS**

**Study Design**

There were two UC San Diego study sites – Altman Clinical and Translational Research Institute (ACTRI) and Exercise and Physical Activity Resource Center (EPARC). ACTRI was the first site and started in August 2019. The decision to add EPARC as an additional site was fortuitously made in January 2020. At the time this was done to increase the volume of subjects seen. However, in March 2020, with the onset of the Covid pandemic, ACTRI became solely a Covid vaccine study location and all other research activities at it were indefinitely paused. Consequently, EPARC took over as the study’s sole UCSD site and outstanding ACTRI participants were transferred to EPARC’s oversight.

**Participants**

The study was advertised on Research Match (a non-profit resource funded by the US National Institutes of Health (NIH) to connect potential participants with researchers), Facebook, Google Ads and via word of mouth. To determine the eligibility of participants, screening was completed over the telephone and via a face-to-face visit at the relevant research site by a study coordinator. The full list of eligibility criteria is below.

*Inclusion Criteria*

1. Signed informed consent.
2. Body mass index (BMI) ≥ 27 kg/m^2^.
3. Males or females. Note females of childbearing potential must have a negative urine pregnancy test before each DXA scan. (As DXA involves a small dose of ionizing radiation). They should agree to follow a contraceptive regimen for the duration of the study period (other than DMPA (Depo-Provera) injections as this causes weight gain).
4. 22-80 years of age inclusive on starting the study. (In order to comply with FDA guidance: https://www.fda.gov/MedicalDevices/DeviceRegulationandGuidance/GuidanceDocuments/ucm089740.htm#s6)
5. Ability and willingness to complete all study visits and procedures; in particular an agreement to engage with: trying to use the device on a daily basis; the hypocaloric diet weight loss program; and this provided weight loss support and mentoring.
6. Agreement not to use prescription, or over the counter, weight loss medications for the duration of the trial.
7. Agreement not to start smoking or vaping tobacco for the duration of the study.
8. Access to Wi-Fi (to connect iPod to internet)

*Exclusion Criteria*

1. History of vestibular dysfunction or other inner ear disease (e.g. significant tinnitus) as indicated by the screening questions.
2. History of bariatric surgery or gastric resection.
3. History of skin breakdown, eczema or other dermatological condition (e.g. psoriasis) affecting the skin behind the ears.
4. History of weight loss device implantation (e.g. VBloc Maestro or Abiliti).
5. Use of a non-invasive weight loss device (e.g. ever used Modius).
6. Hypothyroidism requiring current treatment with levothyroxine (e.g. Levo-T, Synthroid, Thyroxine) (Other thyroid disorder patients on stable treatment for at least 3 months are acceptable).
7. Other endocrinological causes of weight gain (e.g. Cushing’s disease, Cushing’s syndrome or acromegaly)
8. Previous diagnosis of HIV infection or AIDS (HIV is known to cause a vestibular neuropathy which would prevent VeNS from working).
9. Diagnosis of cirrhosis, chronic pancreatitis, or liver, kidney or heart failure.
10. Treatment with prescription weight-loss drug therapy in the 6 months before starting the study.
11. Tobacco smoking (including vaping) in the six months prior to starting and for the duration of the study.
12. Use of marijuana (smoking, vaping or in edible form) more than twice a month on average.
13. Known genetic cause of obesity (e.g., Prader-Willi Syndrome).
14. Body weight change of more than 20% in either direction within the previous year.
15. Following another diet, and/ or current, active member of another organized weight loss program. Unless the participant wishes to discontinue involvement in that diet/ weight loss program and instead participate in the weight loss program provided in this study.
16. Diabetes mellitus (Types 1 & 2).
17. Diagnosis of epilepsy or use of anti-epileptic medication within 6 months of starting the study (e.g. for the treatment of peripheral neuropathy)
18. Chronic (more than a month of daily use) treatment with opioid analgesic drugs within the last 6 months.
19. Regular use (more than twice a week) of anti-histamine medication within the last 6 months.
20. Use of oral or intravenous corticosteroid medication within 6 months of starting the study.
21. Use of beta-blockers within 3 months of starting the study.
22. Current alterations in treatment regimens of anti-depressant medication for whatever reason (including tricyclic antidepressants) (Note: stable treatment regimen for prior 6 months acceptable).
23. An active diagnosis of cancer.
24. A myocardial infarction within the preceding year.
25. A history of stroke or severe head injury (as defined by a head injury that required a craniotomy or endotracheal intubation). (In case this damaged the neurological pathways involved in vestibular stimulation).
26. Presence of permanently implanted battery powered medical device or stimulator (e.g., pacemaker, implanted defibrillator, deep brain stimulator, vagal nerve stimulator etc.).
27. Psychiatric disorders (including untreated severe depression, schizophrenia, substance abuse, eating disorder etc.)
28. Current participant in another weight loss study or other clinical trial.
29. Have a family member who is currently participating in this study.
30. Weight over 350 pounds at UU, TDE and CTRI site (as this is the weight limit of the DXA scanner) or a weight over 500 pounds at the EPARC site (as this is the weight limit of the DXA scanner).
31. Pregnancy.

**Procedures**

Study coordinators demonstrated how to effectively apply the device, clean the skin behind each ear over the mastoid process with an alcohol wipe, place an electrode there, and how to attach the study device to these electrodes. They also showed participants how to switch on their device via a button and adjust the stimulation level either via buttons, or Bluetooth using the study app on the iPod. Both the active and sham device were recharged using a micro-USB Cable, with software controls to ensure the device would not deliver stimulation while charging. The device recorded usage data, which was uploaded when the iPod connected to Wi-Fi. If a participant’s usage over the previous two weeks was below 9 hours, then the study team was automatically alerted so they could encourage usage.

**Sham Device Design**

Designing a sham device that conveyed an adequate degree of verisimilitude so as to maintain blinding was a process that required considerable effort. The design of the sham device was inspired by the sham devices used for studies on transcranial direct current stimulation (tDCS). This is appropriate as vestibular nerve stimulation (VeNS) can be viewed as specialized variant of tDCS that targets the vestibular system rather than the cerebral cortex. Careful consideration was given to generate a sham device with an appropriate degree of authenticity, to maintain participant blinding, while simultaneously not, inadvertently, causing significant vestibular system stimulation in the control subjects.

It has been observed that, due to participants accommodating to the current, the tingling or itching sensations perceived on the skin during tDCS typically last about 30 seconds or so before abating^1,2^. This led to Gandiga et al. to carry out a study in which both the tDCS and sham were increased in a ramp like fashion over about 10 seconds, however while the tDCS continued for 20 minutes, the sham was then turned off at the 30 second timepoint.^3^ Thus, the sham device still delivered the initial skin sensation, while at the same time not providing prolonged stimulation. The authors reported that neither stroke patients nor healthy controls could distinguish between genuine tDCS and sham stimulation, even after experiencing both conditions.

The sham device deployed in our study follows this pattern by applying some stimulation to a participant for a limited period (30 seconds), before tapering down to zero over a further 20 seconds, thus creating the impression of an active device. Moreover, participants were informed in the informed consent document that it is normal for the sensation to be stronger initially on first turning on the device and then for it to, quite quickly, become less noticeable. It elaborates that this is because they “accommodate” to the stimulation. The sham stimulus is applied as a positive and negative bias square wave signal to the participant (just like the active device), but with a frequency of 0.8Hz.

The decision for the sham to deliver at a frequency of 0.8Hz during each stimulation session was made to reduce the likelihood of a vestibulosympathetic response, specifically as measured by muscle sympathetic nerve activity (MSNA). This is because MSNA has been directly linked to weight loss^4^, and it would seem likely that any putative vestibular modulation of body mass composition would, at least in part, be mediated via the sympathetic nervous system. However, the 0.8Hz delivery in the sham device is less likely to affect MSNA than the 0.5Hz delivery in the active device^5,6^.

VeNS poses the additional challenge, over tDCS, when designing a sham that subjects may not only be aware of skin tingling, but also of vestibular stimulation. The sham device addresses this by not only temporarily providing the sensation of skin tingling but also vestibular stimulation, without as significantly modulating MSNA (as it is at 0.8Hz rather than the 0.5Hz frequency of the active device). This should increase the likelihood that sham participants believe they have been allocated an active device. Moreover, three other factors are also pertinent when considering the verisimilitude of the sham device. First, in a manner analogous to the cutaneous sensations, the perceptual salience of the vestibular sensation may decrease with time^7^. Second, all the study subjects will be naïve to GVS. Third, the sham stimulation can be either a positive or negative bias DC electrical current with a max output of 1mA. The bias of the sham signal is determined at random by the device’s embedded software. This was so that the subjects would experience varied sensations when they use the device. Regardless, the stimulation was only active when the participant starts a session and lasts for a total of 50 seconds.

In order to have the sham device cause salient skin sensations during these 50 seconds, the current delivered by the device was, unbeknownst to participants, set in bands as follows:

Level 0 No stimulation applied 0mA

Level 1 – 3 Stimulation applied 0.5mA

Level 4 – 7 Stimulation applied 0.7mA

Level 8 – 10 Stimulation applied 1mA

However, when decreasing the stimulation the sham device would reduce in 1mA increments. This level of control allowed participants to decrease the stimulation if they found it too intense but maintain a level of stimulation that gave the impression that the device was active.

After the initial 50 seconds sham devices would no longer output any significant stimulation for the duration of the setting. Participant would still see activity on the study app and be able to modify the apparent stimulation level via this app, or via the buttons on the device. When doing so the device would appear to the participant to be operating as normal, including the single beep that acknowledges a change in the stimulation level (all beeps and light functions are identical between the sham and active device).

At the 12, 24, 36 and 48-minute timepoints the sham device would confirm correct electrode attachment. It would do so by delivering an imperceptible 0.03mA pulse for 10 seconds. If a disconnection is detected then the app will alert the participant to reposition the electrodes, thus additionally improving its verisimilitude. Only once the device has been power cycled (turned off and on again), or after pausing the stimulation for more than 5 minutes, would the 50 second placebo stimulation period begin again. Externally sham and active devices could only be distinguished by a unique serial number that was known only to the Sponsor’s manufacturing team.

**Figure 1: Active Device Waveform**


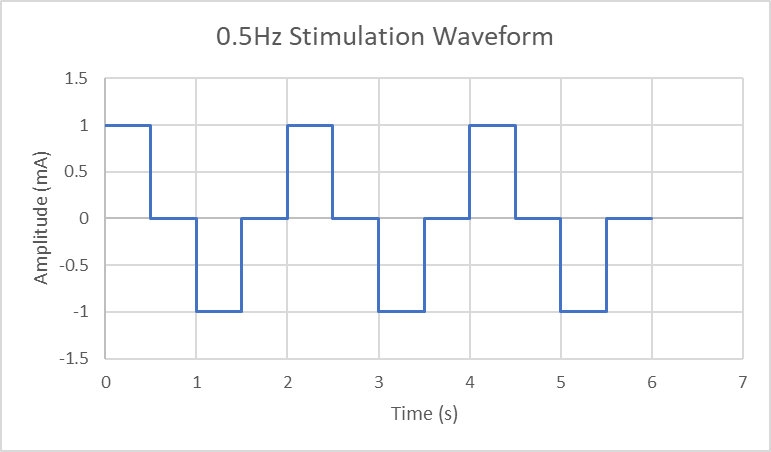


**Lifestyle Modification Program**

At baseline a study dietitian would calculate each participant’s basal metabolic rate (BMR) using the Harris-Benedict equation, then calculate total daily energy expenditure by multiplying the BMR by 1.3^8^. They then prescribed a 600kcal deficit hypocaloric diet for each participant. Similar hypocaloric diets are the standard lifestyle modification programs used in trials of weight loss medications^9-13^. The dietitian also provided sample meal plans with weight loss and weight maintenance tips. At 3 months a dietitian reviewed each participant’s weight and adherence to their diet plan and provided ongoing counselling.

Participants were additionally provided with dietary support by a company called Clinical Trial Mentors (CTM) <https://ctmentors.com/>. The purpose was to support the participants in adhering to their dietitian prescribed hypocaloric diet. Participants were invited to attend a private discussion via a messaging service or a face-to-face call with a mentor once per week over Zoom, WhatsApp, or Facebook. To monitor progress the participants weighed themselves in advance of each session and informed their mentor of this weight. These weights were to guide this interaction and were not collected as part of the study data. Participants were asked to attend at least 75% of these discussions.

There was no mandatory exercise regimen as part of the study. This is because exercise appears to be a less important factor in generating weight loss than diet^14^. This observation may in part be due to compensatory behavioural adaptations (e.g., eating more), but also the fact that energy expenditure is constrained, meaning that increasing levels of exercise do not, in fact, lead to ever increasing levels of total energy expenditure. Rather, due to a compensatory fall in other areas of energy expenditures, total energy expenditure plateaus with increasing exercise^15^. Nonetheless, exercise has multiple health benefits and participants were provided with a PDF of the Physical Activity Guidelines for Americans from the US Department of Health (<https://health.gov/sites/default/files/2019-09/paguide.pdf>). They were told that chapters 4 to 6 and chapter 8 of these were the most informative for them to read. These are the chapters that respectively detail the guidelines for: Active Adults; Active Older Adults; Safe and Active; and Taking Action: Increasing Physical Activity Levels of Americans. They were also given access to an Activity Tracker Pedometer app on the study iPod to count their daily steps. The pedometer app data was not used in the analysis.

**Outcomes**

Whole-body DXA scans were undertaken at the clinical sites. EPARC, ACTRI and TDE used the Hologic Discovery system, and UU used the GE Healthcare Lunar machine. Computed tomography (CT) scanning is considered a gold standard (together with magnetic resonance imaging) in measuring VAT and the measurements of VAT estimated by both the Hologic and GE systems have been shown to be highly correlated with CT.^16,17,18^ Moreover, DXA only exposes patients to a tiny fraction of the ionising radiation that CT does.

In total 25 out of a total of 120 follow up DXA scans were obtained on the GE DXA and the rest were done on the Hologic machine. As well as being highly correlated with CT estimates of VAT as discussed, Hologic and GE VAT measures are reported as being highly correlated with one another, with published coefficients of correlation of 0.93 and 0.97.^19,20^ Notably, all participants who received subsequent DXA scans were scanned using the same type of system as at baseline. Total body fat, visceral adipose tissue, trunk fat, lean muscle mass and bone mineral content measurements were calculated. At the ACTRI site 18 participants mistakenly underwent a 3-month DXA scan (they were scheduled to be only conducted at baseline and 6 months). This proved fortuitous as in almost all cases these participants missed their 6-month DXA due to the Covid pandemic. After informing the UCSD IRB and gaining its permission these data were incorporated into the statistical analysis for missing data.

Body Mass Index (BMI) was calculated as weight (kg) divided by height squared (m^2^). Standing height was measured to the nearest 0.1 cm without shoes. Waist circumference was measured using a tape measure placed around the waist in the horizontal/transverse plane at the height of the navel. (This was not done at the UK site due to local concerns). To monitor change in cardiovascular risk factors, fasting venous blood samples were taken for lipid profile, high sensitivity CRP, glycosylated haemoglobin, and glucose.

Dietary intake was assessed using the USDA multiple-pass 24-hour recall method. The method was adapted to use three distinct passes and collected information about the participant’s food intake during the preceding weekday and weekend day (two-day 24-hour recall) to better reflect the overall diet. The study dietitians conducted an interview with the participants, which involved: (1) asking the participants to list all the foods and beverages consumed with no interruptions; (2) probing about frequently forgotten foods; and (3) asking for more details on the description of the foods and amounts eaten aided by the Portion Photos of Popular Foods guide (<https://culinarynutritionpublishing.com/portion-photos-of-popular-foods/>). The dietary data was analysed using a nutrition analysis software package designed by Nutritics (<https://www.nutritics.com/p/clinical>). Mean total energy intake (kcal) was used for analysis.

The Duke University impact of weight on quality of life (IWQoL) questionnaire was licensed to assess obesity-specific quality of life.^21^ It is a widely used instrument in weight-loss interventions. A total score was calculated for each participant, with higher scores representing a better quality of life. During the study device usage compliance was measured using the pre-installed Modius study app. When the iPod was connected to Wi-Fi, the data logs were transferred to secure cloud storage. The duration of usage (minutes) and number of sessions logged were used to calculate the mean number of weekly sessions. The data recorded for each session were: duration of usage; average intensity (current) selected by the participant; and average electrical impedance. However, to ensure blinding only sessions logged and duration of usage data were accessible to study personnel during the trial. After the onset of the Covid-19, in April 2020 a questionnaire was formulated to determine the impact of the pandemic on the participants’ diet and physical activity. This was distributed to participants via the Modius app.

**Audiology Assessments**

The FDA requested during discussions before starting the trial that participants undergo a hearing test and otoscope examination of the ear canal at 0 and 6-months. The FDA allowed a two-week window after the subjects were seen and commenced device usage for these tests to be carried out. There is no suggestion from any of the literature looking at the safety of vestibular nerve stimulation that hearing, or the ear canal are adversely affected.^22,23^ And that a study specifically looking at the impact of GVS on cochlear function showed no effect.^24^

Rather intriguingly obesity and a sedentary lifestyle have been linked with increased tinnitus symptoms.^25,26^ Why this is so is unclear, although it may be due to Western-style diets, which are high in fat and simple carbohydrates, with low antioxidant intake and increased systemic inflammation.^25^ Conversely, low glycemic index diets and increased physical activity show potential in alleviating tinnitus symptoms.^25,26^ A study of 63 obese tinnitus patients demonstrated that dietary and physical activity interventions, alone or combined, significantly reduced tinnitus severity, depression, and body weight while improving quality of life.^26^ These findings underscore the complex relationship between obesity and hearing.

In the UK site the ear canal assessment and hearing test was done by a professional audiologist placing a pair of insert headphones who carried out a standard hearing test to determine if hearing loss is present, and if so, how much and of what type. A plot of frequency versus amplitude sensitivity threshold for each ear was generated. The speech recognition threshold and word recognition score were also obtained. All testing was completed in a quiet booth to avoid extraneous noise.

In the USA, prior to the hearing assessment, the outer canals of the study participant’s ears were examined with an otoscope, photos captured and reviewed by the PI, Dr Erik Viirre MD PhD who is a clinical neuro-otologist at UC San Diego Health. At the USA sites subjects received a hearing assessment using the AMTAS Flex system, which is made by Grason-Stadler (<https://www.grason-stadler.com/products/audiometers/gsi-amtas-flex>). This performs an assessment based on pure tone audiography and produces a plot of hearing sensitivity at the same frequencies assessed in a regular hearing test. This was carried out with the participants wearing headphones in a quiet room and coordinators who oversaw it were first trained and signed off by EV, who is a clinical neuro-otologist. If any concern of an abnormal finding was raised during the AMTAS Flex assessment, then the participant was sent for formal audiology assessment.

**Figure 2: Copy of Adverse Event Questionnaire – assessed for both during and after usage at 1, 3 and 6 months.**

1. Headache

| Never | Almost never | Sometimes | Almost every time | Every time |
| --- | --- | --- | --- | --- |

1. Pain behind ears

| Never | Almost never | Sometimes | Almost every time | Every time |
| --- | --- | --- | --- | --- |

1. Vertigo/dizziness

| Never | Almost never | Sometimes | Almost every time | Every time |
| --- | --- | --- | --- | --- |

1. Blurred Vision

| Never | Almost never | Sometimes | Almost every time | Every time |
| --- | --- | --- | --- | --- |

1. Nausea

| Never | Almost never | Sometimes | Almost every time | Every time |
| --- | --- | --- | --- | --- |

1. Fatigue/tiredness

| Never | Almost never | Sometimes | Almost every time | Every time |
| --- | --- | --- | --- | --- |

1. Tinnitus (ringing sound no one else can hear):

| Never | Almost never | Sometimes | Almost every time | Every time |
| --- | --- | --- | --- | --- |

1. Seizures:

| Never | Almost never | Sometimes | Almost every time | Every time |
| --- | --- | --- | --- | --- |

1. Other: (Please specify) ____________________________________

| Never | Almost never | Sometimes | Almost every time | Every time |
| --- | --- | --- | --- | --- |

**Table 1**: Schedule of Study Assessments and Events

| **Visit ID** | **Visit 1** | **Visit 2** | **Visit 3** |
| --- | --- | --- | --- |
| **Visit Description** | **Screening/ Baseline** | **Month 3** | **Month 6**  **End of trial** |
| **Visit Window (Months ± 7 Days)** | **0 Months** | **3 Months** | **6 Months** |
| Inclusion/Exclusion Questionnaire | X |  |  |
| Diabetes screening – finger prick | X |  |  |
| ThyroChek test for hypothyroidism (CLIAwaived Inc) | X |  |  |
| Urine pregnancy test (if applicable) | X | X | X |
| Record demographic details | X |  |  |
| Record medications or change in medications | X |  | X |
| Randomise into study/ give allocated device and supplies/ train how to use | X |  |  |
| Check skin behind ears | X | X | X |
| Weight (underwear and gown) | X | X | X |
| Height (no shoes) | X |  |  |
| Waist and hip measurements (underwear and gown) | X* |  | X* |
| Fasting glucose | X |  | X |
| Glycosylated haemoglobin (HbA1c) | X |  | X |
| High sensitivity CRP | X |  | X |
| Lipid panel | X |  | X |
| Heart rate and blood pressure | X |  | X |
| Quality of life survey (Duke University IWQOL) | X |  | X |
| Adverse event questionnaire | X^0^ | X | X |
| DXA Scan (whole body scan) | X |  | X |
| Hypocaloric diet counseling | X^1^ | X^1^ |  |
| Two-day 24-hour dietary recall | X^2^ | X^2^ | X^2^ |
| Food Frequency Questionnaire | X^2^ |  |  |
| Modifiable Activity Questionnaire | X^2^ |  | X^2^ |
| Hearing test/ otoscope examination | X |  | X |
| Blinding assessment by subject | X^3^ |  | X^3^ |
| Blinding assessment by coordinator |  |  | X |

^*^ This measurement was only performed at the USA sites.

^0^ At 1-month timepoint the Adverse Event Questionnaire was done via telephone or email. At 6-month timepoint the Adverse Event Questionnaire was completed in person.

^1^ Dietary counseling from a research dietitian with emphasis on weight loss and healthy eating via a hypocaloric diet.

^2^ Research dietitian had the subject complete this activity.

^3^ This was also be performed at 0-Month and 1-Month timepoints collected via the study app and at 6-Months in person at the clinical site. At 0-Month the study app notification was displayed after the first session of using the study device

^4^ If the participant completed the study to Visit 3 (at 6 months), then they were given the iPod they used throughout as a gift.

**Statistical Analyses**

**Power Calculation**

Simulation assumptions were informed by our pilot data, as follows:^27^

- 90kg median baseline weight in both groups (log-normal distribution with CV=24%)

- 6.6% mean percent weight loss in the active group at 6 months (SD 3.9%),

- 3.1% mean percent weight loss in the control group at 6 months (SD 3.9%)

- correlation between baseline weight and weight loss at 6 months of 0.4.

**Primary Efficacy Criteria**

The study would be deemed to have met the primary efficacy criteria if this statistical hypothesis test was rejected:

- There is no significant difference between the mean percentage weight loss of participants using the active device and the sham device, i.e., 𝑥̅_𝑣_ - 𝑥̅_𝑠_ >= 0, where 𝑥̅_𝑣_ and 𝑥̅_𝑠_ are the mean percentage weight loss of participants using the active device and sham device respectively.

And moreover, if these two numerical criteria were achieved:

- The difference between the mean percentage weight loss of participants using the active device and the sham device is at least 2% as observed from the confidence intervals, (i.e., equivalent to assessing the hypothesis 𝑥̅_𝑣_ - 𝑥̅_𝑠_ ≥ 2).
- The lower bound of the 95% confidence interval of the response rate in the active device group exceeds 50%, with response defined as a loss of at least 5% of body weight i.e., 𝑝𝑣 − ½ (𝐶𝑢 – 𝐶𝐿) ≥ 50%, where 𝑝𝑣 is the proportion of participants who lose at least 5% body weight using the active device. CL and CU are the lower and upper bounds of the 95% confidence interval.

Prior to performing statistical comparisons, the continuous efficacy endpoints were evaluated for conformance to parametric assumption by assessing their distribution. A per protocol population analysis was also conducted and defined as all participants who did not violate the protocol in terms of a major deviation. To be included in the per protocol population, subjects must use the device for at least an average of three and a half hours a week for at least four of the first 6 months.

**Secondary Outcomes**

As discussed in the main paper, change in VAT at 6 months was the first labelling claim associated secondary endpoint. As measured by the whole body DXA scan, VAT loss, was considered successful if significance was declared with the following statistical hypothesis:

- There is no significant difference between the mean percentage VAT loss of participants using the active device and the sham device, i.e., 𝑥̅_𝑣_ - 𝑥̅_𝑠_ = 0, where 𝑥̅_𝑣_ and 𝑥̅_𝑠_ are the mean percentage VAT loss of participants using the active device and sham device respectively.

Due to the Covid-19 pandemic 31 participants in the active and 32 in the sham group were unable to attend for DXA scans at 6-months. Of those participants who had a DXA scan at baseline and 6-months, VAT measurements were missing for 16 participants (active n=11 and control n=5) due to their hip width not meeting the parameters of the machine for this specific measurement. One participant in the active group was missing a DXA scan at baseline. As described, missing 6-month data for these participants were imputed for the ITT analysis.

The second labelling claim associated secondary endpoint to be tested was LDL. A decrease in LDL was considered successful if significance was declared with the following statistical hypothesis:

- There is no significant difference between the mean percentage change from baseline in LDL of participants using the active device and the sham device, i.e. 𝑥̅_𝑣_ − 𝑥̅_𝑠_ = 0, where 𝑥̅_𝑣_ and 𝑥̅_𝑠_ are the mean percentage change from baseline in LDL of participants using the active device and sham device respectively.

The third labelling claim associated secondary endpoint to be tested was fat loss. An increase in fat loss, as determined by the whole body DXA scan, was considered successful if significance was declared with the following statistical hypothesis:

- There is no significant difference between the mean percentage fat loss of participants using the active device and the sham device, i.e., 𝑥̅_𝑣_ − 𝑥̅_𝑠_ = 0, where 𝑥̅_𝑣_ and 𝑥̅_𝑠_ are the mean percentage fat loss of participants using the active device and sham device respectively.

The fourth labelling claim associated secondary endpoint to be tested was lean muscle mass. An increase in lean muscle mass, as determined by the whole body DXA scan, was considered successful if significance was declared with the following statistical hypothesis:

- There is no significant difference between the mean percentage change in lean muscle mass of participants using the active device and the sham device, i.e., 𝑥̅_𝑣_ − 𝑥̅_𝑠_ = 0, where 𝑥̅_𝑣_ and 𝑥̅_𝑠_ are the mean percentage change in lean muscle mass of participants using the active device and sham device respectively.

The fifth labelling claim associated secondary endpoint to be tested was the atherogenic index. A decrease in the atherogenic index, as determined by the ratio of total cholesterol to HDL, was considered successful if significance was declared with the following statistical hypothesis:

- There is no significant difference between the mean change from baseline for the ratio of total cholesterol to HDL of participants using the active device and the sham device, i.e., 𝑥̅_𝑣_ −𝑥̅_𝑠_ = 0, where 𝑥̅_𝑣_ and 𝑥̅_𝑠_ are the mean change from baseline in the ratio of total cholesterol to HDL of participants using the active device and sham device respectively.

The sixth labelling claim associated secondary endpoint to be tested was systemic inflammation. A decrease in systemic inflammation, as measured by high-sensitivity C-reactive protein, was considered successful if significance was declared with the following statistical hypothesis:

- There is no significant difference between the mean change from baseline for the high-sensitivity C-reactive protein of participants using the active device and the sham device, i.e., 𝑥̅_𝑣_ − 𝑥̅_𝑠_ = 0, where 𝑥̅_𝑣_ and 𝑥̅_𝑠_ are the mean change from baseline in the high-sensitivity C-reactive protein of participants using the active device and sham device respectively.

The seventh labelling claim associated secondary endpoint that was tested at 6 months is total calorie intake. A decrease in total calorie intake, as measured by the two-day 24-hour dietary recall, was considered successful if significance was declared with the following statistical hypothesis:

- There is no significant difference between the mean change from baseline for the total calorie intake of participants using the active device and the sham device, i.e., 𝑥̅_𝑣_ − 𝑥̅_𝑠_ = 0, where 𝑥̅_𝑣_ and 𝑥̅_𝑠_ are the mean change from baseline of the total calorie intake of participants using the active device and sham device respectively.

The eighth labelling claim associated secondary endpoint that was assessed at 6 months is quality of life. An increase in quality of life, as determined using the IWQoL questionnaire, was considered

successful if significance was declared with the following statistical hypothesis:

- There is no significant difference between the mean change from baseline for the total QOL score of participants using the active device and the sham control, i.e., 𝑥̅_𝑣_ − 𝑥̅_𝑠_ = 0, where 𝑥̅_𝑣_ and 𝑥̅_𝑠_ are the mean change from baseline in total QOL scores of participants using the active device and sham device respectively.

The baseline characteristics of each treatment group were compared descriptively, with summary statistics provided. For the primary outcome and secondary outcomes, the differences between treatment groups were assessed using a linear model controlling for covariates: gender and baseline values (of the outcome measure). The least square (LS) mean difference between groups (primary and secondary outcomes) were assessed for a statistically significantly difference from 0 i.e., p-value <0.05.

**Blinding Assessment**

An approach outlined by James et al. was used for the blinding analysis, where an index was created based on a modification of a Kappa statistic to incorporate the “Do not know” category.^28^ The index ranges from 0 to 1 and provides an indication of how good the blinding was, where 0 implies complete lack of blinding and 1 implies complete blinding. The weight assigned to the three response options were 0 for a correct guess, 1 for a “Do not know” response and 0.75 for an incorrect guess.

For the blinding analysis a table of the actual treatment received against the perceived treatment received showing the numbers and proportions of subjects falling into each of the six categories was produced alongside the index of blindness where a result greater than 0.5 indicates a result better than that obtained by random choices. The 95% asymptotic confidence intervals have been included with the interpretation that a suitable level of blindness has been achieved if the lower bound of the interval is at or above 0.5.

**Additional Results**

**Figure 3** is the CONSORT Participant flowchart. The active group completed on average 6.6 sessions per week, while the sham group completed 5.6 sessions (p=0.056). Device compliance rate was the same in both groups, with 44% of participants adhering to the prescribed dosage (minimum of 5 hours/week for 6 months). Mentor support usage data are presented in **Table 2** below. 52% of participants in the active group and 57% of the participants in the sham group had contact with a study mentor throughout the study. Changes in cardiovascular medication and medication that has the potential to impact weight status were comparable between the randomisation groups as shown in **Table 3**. Figures 4 and 5 are waterfall plots (per-protocol population) showing each participant’s percentage change in total body fat and VAT from baseline to 6 moths by randomisation group. Descriptive summaries of exploratory secondary outcomes according to randomisation group are shown in **Table 4**. The active group had notable changes from baseline to 6 months for waist circumference. The blinding assessment results for the participants and study coordinators are shown in **Table 5**. An adequate level of blinding was maintained throughout the trial. The Covid-19 Pandemic survey results are presented in **Table 6** below. A total of 178 participants (active n=90 and sham n=88) completed the Covid-19 questionnaire via the study app.

**Figure 3: CONSORT participant flowchart**

Assessed for eligibility (n= 317)

Excluded (n=76)

♦  Not meeting inclusion criteria (n= 76)

ITT primary outcome analysis (n=117)
♦ Excluded from analysis (give reasons) (n=0)

Lost to follow-up (give reasons) (n=3)

Discontinued intervention (give reasons) (n=23)

AE (n=7), low usage (n=4), medication change (n=1), voluntary drop (n=9), pregnancy (n=1) & unknown (n=1)

Allocated to Modius Lean Device Group (n=117)

♦ Received allocated intervention (n=117)

♦ Did not receive allocated intervention (give reasons) (n=0)

Lost to follow-up (give reasons) (n=6)

Discontinued intervention (give reasons) (n=29)

AE (n=7), joined another lifestyle program (n=1), low usage (n=8), non-compliance (n=2), voluntary drop (n=9) & unknown (n=2)

Allocated to Sham Device Group (n=124)

♦ Received allocated intervention (n=124)

♦ Did not receive allocated intervention (give reasons) (n=0)

ITT primary outcome analysis (n=124)
♦ Excluded from analysis (give reasons) (n=0)

## Allocation

## Analysis

## Follow-Up

Randomized (n=241)

## Enrollment

CONSORT diagram showing the flow of participants through the trial and analysed for primary outcome at 6 months.

**Table 2: Mentor support Group Usage**

|  |  | **Active device** | **Sham device** |
| --- | --- | --- | --- |
|  |  | **(n=116)** | **(n=122)** |
|  |  | **n (%)** | **n (%)** |
| **Total no. of responses** |  | 2784 | 2893 |
| **Total no. of hours spent per week, n(%)** | No usage | 1336 (48) | 1246 (43) |
|  | 0-2 hours | 917 (33) | 883 (31) |
|  | 2-4 hours | 241 (9) | 349 (12) |
|  | 4-6 hours | 205 (7) | 252 (9) |
|  | Over 6 hours | 85 (3) | 163 (6) |

Number of hours of mentor support usage was reported weekly per subject. Percentages are calculated based on: (Frequency / Total Number of Responses) * 100

**Table 3: Concomitant medication: cardiovascular adjustments and weight gain causality**

| **Medication** |  | **Active device** | **Sham device** |
| --- | --- | --- | --- |
|  |  | **(n=117)** | **(n=124)** |
|  |  | **n (%) [m]** | **n (%) [m]** |
| **Cardiovascular Medication** | Increase | 1 (2) [2] | 4 (3) [4] |
|  | Decrease | 1 (1) [1] | 3 (2) [4] |
|  | No change | 24 (21) [36] | 28 (23) [40] |
| **Medication (known to cause weight gain)** |  | 34 (29) [47] | 42 (34) [63] |
| **Medication (known to cause weight loss)** |  | 23 (20) [28] | 29 (23) [36] |

Treatment adjustment and causality assessment made by suitably qualified members of study team. n = number of subjects with a medication, m = number of medication instances. Note, a subject may be included in more than one category.

**Table 4: Descriptive summaries of exploratory secondary outcomes according to randomisation group**

|  |  | **Active Device** |  | **Sham Device** |  |
| --- | --- | --- | --- | --- | --- |
| **Endpoints** |  | **Baseline** | **6 month** | **Baseline** | **6 month** |
|  |  | **Mean (SD)** |  | **Mean (SD)** |  |
| **Dietary** |  |  |  |  |  |
|  | Carbohydrates (g) | 191.68 (79.98) | 158.55 (69.89) | 188.01 (84.25) | 178.29 (159.16) |
|  | Fat (g) | 78.73 (38.94) | 71.89 (28.72) | 78.51 (37.88) | 72.87 (68.38) |
|  | Saturated fat (g) | 26.11 (14.82) | 24.73 (12.76) | 27.16 (15.16) | 25.97 (28.76) |
|  | Protein (g) | 84.90 (35.95) | 80.01 (25.03) | 85.80 (34.00) | 78.54 (23.69) |
|  | Fibre (g) | 19.31 (7.83) | 18.25 (6.57) | 19.54 (8.99) | 18.62 (9.23) |
|  | Alcohol (g) | 23.67 (17.90) | 25.02 (23.93) | 26.38 (20.50) | 21.63 (20.52) |
| **Cardiovascular risk** |  |  |  |  |  |
|  | Fasting glucose (mg/dL) | 92.46 (9.79) | 94.95 (12.22) | 93.24 (10.30) | 92.72 (9.14) |
|  | HbA1c (%) | 5.49 (0.37) | 5.40 (0.81) | 5.49 (0.39) | 5.50 (0.37) |
|  | Total cholesterol (mg/dL) | 199.48 (42.49) | 198.66 (42.0) | 196.93 (44.48) | 193.35 (37.07) |
|  | LDL cholesterol (mg/dL) | 120.21 (29.40) | 119.35 (26.80) | 120.91 (37.75) | 118.68 (30.74) |
|  | VLDL cholesterol (mg/dL) | 22.23 (9.50) | 22.43 (12.55) | 24.52 (11.42) | 21.51 (8.73) |
|  | HDL cholesterol (mg/dL) | 54.35 (16.03) | 52.87 (14.66) | 51.15 (12.62) | 53.27 (13.30) |
|  | Triglycerides (mg/dL) | 129.48 (166.00) | 141.40 (232.58) | 125.54 (65.86) | 106.88 (44.37) |
|  | Systolic blood pressure (mmHg) | 129.9 (17.26) | 128.3 (20.85) | 126.6 (16.88) | 122.6 (15.31) |
|  | Diastolic blood pressure (mmHg) | 85.4 (11.07) | 85.6 (11.46) | 82.0 (10.10) | 81.3 (8.89) |
|  | Heart rate (bpm) | 70.8 (9.99) | 71.1 (10.29) | 69.6 (10.66) | 68.6 (10.66) |
| **Body composition** |  |  |  |  |  |
|  | BMI (kg/m^2^) | 36.9 (6.68) | 36.6 (6.94) | 35.9 (5.78) | 34.8 (6.25) |
|  | Waist circumference (cm) | 105.2 (14.75) | 99.8 (11.44) | 105.9 (12.16) | 101.3 (11.27) |
|  | Hip Circumference (cm) | 120.8 (12.99) | 120.3 (11.37) | 121.9 (12.42) | 117.7 (13.03) |
|  | Waist Hip Ratio | 0.87 (0.09) | 0.83 (0.08) | 0.87 (0.08) | 0.87 (0.10) |

Includes available data only.

**Table 5: Blinding assessment at baseline, 1 month and 6 months according to randomisation group**

| **Blinding assessment** | **Assessment time** | **Perceived device** | **Active**  **device (n=116)**  **n (%)** | **Sham device (n=122)**  **n (%)** | **Blinding index (95% Cis)** |
| --- | --- | --- | --- | --- | --- |
| **Participant** | Baseline | Active | 42 (36.2) | 33 (27.0) | 0.75 (0.70, 0.80) |
|  |  | Sham | 5 (4.3) | 15 (12.3) |  |
|  |  | Don’t know | 67 (57.8) | 70 (57.4) |  |
|  |  | Missing | 2 (1.7) | 4 (3.3) |  |
|  | 1 month | Active | 47 (40.5) | 33 (27.0) | 0.58 (0.52, 0.65) |
|  |  | Sham | 17 (14.7) | 42 (34.4) |  |
|  |  | Don’t know | 41 (35.3) | 34 (27.9) |  |
|  |  | Missing | 11 (9.5) | 13 (10.7) |  |
|  | 6 month | Active | 30 (25.9) | 13 (10.7) | 0.57 (0.50, 0.64) |
|  |  | Sham | 27 (23.3) | 47 (38.5) |  |
|  |  | Don’t know | 32 (27.6) | 29 (23.8) |  |
|  |  | Missing | 27 (23.3) | 33 (27.0) |  |
| **Trial Coordinator** | 6 month | Active | 31 (26.7) | 27 (22.1) | 0.57 (0.50, 0.64) |
|  |  | Sham | 34 (29.3) | 45 (36.9) |  |
|  |  | Don’t know | 21 (18.1) | 17 (13.9) |  |
|  |  | Missing | 30 (25.9) | 33 (27.0) |  |

**Table 6: Covid-19 Survey Results**

|  | **Decrease** | | **Stayed the same** | | **Increase** | |
| --- | --- | --- | --- | --- | --- | --- |
| **Questionnaire item** | **Active**  **n(%)** | **Control**  **n(%)** | **Active**  **n(%)** | **Control**  **n(%)** | **Active**  **n(%)** | **Control**  **n(%)** |
| Change in appetite | 17 (19) | 9 (10) | 24 (27) | 26 (30) | 49 (54) | 53 (60) |
| Length of time to feel full | 17 (19) | 19 (22) | 34 (38) | 29 (33) | 39 (43) | 40 (45) |
| Making more negative food choices | 24 (27) | 25 (28) | 19 (21) | 20 (23) | 37 (41) | 43 (49) |
| Change in physical activity levels | 58 (64) | 54 (61) | 10 (11) | 9 (10) | 22 (24) | 25 (28) |

APPENDIX REFERENCES

1. Nitsche MA, Liebetanz D, Lang N, Antal A, Tergau F, Paulus W. Safety criteria for transcranial direct current stimulation (tDCS) in humans. *Clin Neurophysiol* 2003; **114**: 2220–2. doi: 10.1016/s1388-2457(03)00235-9.
2. Paulus W. Transcranial direct current stimulation (tDCS). *Suppl Clin Neurophysiol* 2003; **56**: 249–54. doi: 10.1016/s1567-424x(09)70229-6.
3. Gandiga PC, Hummel FC, Cohen LG. Transcranial DC stimulation (tDCS): a tool for double-blind sham-controlled clinical studies in brain stimulation. *Clin Neurophysiol* 2006; **117**: 845–50. doi: 10.1016/j.clinph.2005.12.003.
4. Straznicky NE, Grima MT, Sari CI et al. Neuroadrenergic dysfunction along the diabetes continuum: a comparative study in obese metabolic syndrome subjects. *Diabetes* 2012; **61**: 2506–16. doi: 10.2337/db12-0138.
5. Grewal T, James C, Macefield VG. Frequency-dependent modulation of muscle sympathetic nerve activity by sinusoidal galvanic vestibular stimulation in human subjects. *Exp Brain Res* 2009; **197**: 379–86. doi: 10.1007/s00221-009-1926-y.
6. Macefield VG, James C. Superentrainment of muscle sympathetic nerve activity during sinusoidal galvanic vestibular stimulation. *J Neurophysiol* 2016; **116**: 2689–94. doi: 10.1152/jn.00036.2016.
7. Fitzpatrick RC, Day BL. Probing the human vestibular system with galvanic stimulation. *J Appl Physiol* 2004; **96**: 2301–6. doi: 10.1152/japplphysiol.00008.2004.
8. Harris JA, Benedict FG. A biometric study of human basal metabolism. *Proc Natl Acad Sci USA* 1918; **4**: 370–3.
9. Sjöström L, Rissanen A, Andersen T, et al. Randomised placebo-controlled trial of orlistat for weight loss and prevention of weight regain in obese patients. European Multicentre Orlistat Study Group. *Lancet* 1998; **352**: 167–72. doi: 10.1016/s0140-6736(97)11509-4.
10. Smith SR, Weissman NJ, Anderson CM, et al. Behavioral Modification and Lorcaserin for Overweight and Obesity Management (BLOOM) Study Group. Multicenter, placebo-controlled trial of lorcaserin for weight management. *N Engl J Med* 2010; **363**: 245–56. doi: 10.1056/NEJMoa0909809.
11. Allison DB, Gadde KM, Garvey WT, et al. Controlled-release phentermine/topiramate in severely obese adults: a randomized controlled trial (EQUIP). *Obesity (Silver Spring*) 2012; **20**: 330–42. doi: 10.1038/oby.2011.330.
12. Apovian CM, Aronne L, Rubino D, et al. COR-II Study Group. A randomized, phase 3 trial of naltrexone SR/bupropion SR on weight and obesity-related risk factors (COR-II). *Obesity (Silver Spring)* 2013; **21**: 935–43. doi: 10.1002/oby.20309.
13. Wadden TA, Hollander P, Klein S, et al. NN8022-1923 Investigators. Weight maintenance and additional weight loss with liraglutide after low-calorie-diet-induced weight loss: the SCALE Maintenance randomized study. *Int J Obes* 2013; **37**: 1443–51. doi: 10.1038/ijo.2013.120.
14. Melanson EL, Keadle SK, Donnelly JE, Braun B, King NA. Resistance to exercise-induced weight loss: compensatory behavioral adaptations. *Med Sci Sports Exerc* 2013; **45**: 1600–9. doi: 10.1249/MSS.0b013e31828ba942.
15. Pontzer H, Durazo-Arvizu R, Dugas LR, et al. Constrained Total Energy Expenditure and Metabolic Adaptation to Physical Activity in Adult Humans. *Curr Biol* 2016; **26**: 410–7. doi: 10.1016/j.cub.2015.12.046.
16. Kaul S, Rothney MP, Peters DM, et al. Dual-energy X-ray absorptiometry for quantification of visceral fat. *Obesity (Silver Spring)* 2012; **20**: 1313–8. doi: 10.1038/oby.2011.393. Erratum in: *Obesity (Silver Spring)* 2012; **20**: 1544.
17. Micklesfield LK, Goedecke JH, Punyanitya M, Wilson KE, Kelly TL. Dual-energy X-ray performs as well as clinical computed tomography for the measurement of visceral fat. *Obesity (Silver Spring)* 2012; **20**: 1109–14. doi: 10.1038/oby.2011.367.
18. Goldberg EK, Fung EB. Precision of the Hologic DXA in the Assessment of Visceral Adipose Tissue. *J Clin Densitom* 2020; **23**: 664-672.
19. Fan B, Wilson J, Shepherd J, Wu X. How comparable are Hologic and GE-Lunar visceral fat analyses? *J Clin Densitom* 2013; **16**: 263. doi: [10.1016/j.jocd.2013.05.007](http://dx.doi.org/10.1016/j.jocd.2013.05.007)
20. Bennett JP, Fan B, Liu E, Kazemi L, Wu XP, Zhou HD, Lu Y, Shepherd JA. Standardization of dual-energy x-ray visceral adipose tissue measures for comparison across clinical imaging systems. *Obesity (Silver Spring)* 2023; **31**: 2936-2946. doi: 10.1002/oby.23885.
21. Kolotkin RL, Crosby RD, Kosloski KD, Williams GR. Development of a brief measure to assess quality of life in obesity*. Obes Res* 2001; **9**: 102–11.
22. Krystal, A, Zammit GK, Wyatt JK et al. The effect of vestibular Stimulation in a four-hour sleep phase advance model of transient insomnia. *Journal of Clinical Sleep Medicine* 2010; **6**: 315–21.
23. Marshall, M, Zang H, Jasko JG. Therapeutic effectiveness and patient acceptance of a vestibular nerve activation intervention in chronic insomnia. *Medicamundi* 2010; **54**: 89–93.
24. Cevette MJ, Cocco D, Pradhan GN, et al. The effect of galvanic vestibular stimulation on distortion product otoacoustic emissions. *J Vestib Res* 2012; **22**: 17–25. doi: 10.3233/VES-2012-0444.
25. Özbey-Yücel Ü, Uçar A. The role of obesity, nutrition, and physical activity on tinnitus: A narrative review. *Obesity Medicine* 2023; **40**: 100491.
26. Özbey-Yücel Ü, Uçar A, Aydoğan Z, Tokgoz-Yilmaz S, Beton S. The effects of dietary and physical activity interventions on tinnitus symptoms: An RCT. *Auris Nasus Larynx* 2023; **50**: 40–47. doi: 10.1016/j.anl.2022.04.013.
27. McGeoch P, McKeown J, Peterson H, Ramachandran V. Modulation of body mass composition using vestibular nerve stimulation. *bioRxiv* 2017; 087692. doi: <https://doi.org/10.1101/087692>.
28. James KE, Bloch DA, Lee KK, Kraemer HC, Fuller RK. An index for assessing blindness in a multi-centre clinical trial: disulfiram for alcohol cessation--a VA cooperative study. **Stat Med** 1996; 15: 1421–34. doi: 10.1002/(SICI)1097-0258(19960715)15:13<1421.


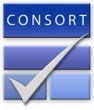
CONSORT 2010 checklist of information to include when reporting a randomised trial*

| Section/Topic | Item No | Checklist item | Reported on page No |
| --- | --- | --- | --- |
| Title and abstract | | | |
|  | 1a | Identification as a randomised trial in the title | 1 |
|  | 1b | Structured summary of trial design, methods, results, and conclusions (for specific guidance see CONSORT for abstracts) | 2 |
| Introduction | | | |
| Background and objectives | 2a | Scientific background and explanation of rationale | 3 & 4 |
|  | 2b | Specific objectives or hypotheses | 4 |
| Methods | | | |
| Trial design | 3a | Description of trial design (such as parallel, factorial) including allocation ratio | 9 |
|  | 3b | Important changes to methods after trial commencement (such as eligibility criteria), with reasons | NA |
| Participants | 4a | Eligibility criteria for participants | 10 & Supplementary information |
|  | 4b | Settings and locations where the data were collected | 9 |
| Interventions | 5 | The interventions for each group with sufficient details to allow replication, including how and when they were actually administered | 11 & Supplementary information |
| Outcomes | 6a | Completely defined pre-specified primary and secondary outcome measures, including how and when they were assessed | 12, 13 & Supplementary information |
|  | 6b | Any changes to trial outcomes after the trial commenced, with reasons | 11 |
| Sample size | 7a | How sample size was determined | 13, 14 & Supplementary information |
|  | 7b | When applicable, explanation of any interim analyses and stopping guidelines | NA |
| Randomisation: |  |  |  |
| Sequence generation | 8a | Method used to generate the random allocation sequence | 10 |
|  | 8b | Type of randomisation; details of any restriction (such as blocking and block size) | 10 |
| Allocation concealment mechanism | 9 | Mechanism used to implement the random allocation sequence (such as sequentially numbered containers), describing any steps taken to conceal the sequence until interventions were assigned | 10 |
| Implementation | 10 | Who generated the random allocation sequence, who enrolled participants, and who assigned participants to interventions | 10 |
| Blinding | 11a | If done, who was blinded after assignment to interventions (for example, participants, care providers, those assessing outcomes) and how | 10 |
|  | 11b | If relevant, description of the similarity of interventions | 10 |
| Statistical methods | 12a | Statistical methods used to compare groups for primary and secondary outcomes | 13, 14, 15 & Supplementary information |
|  | 12b | Methods for additional analyses, such as subgroup analyses and adjusted analyses | 15 |
| Results | | | |
| Participant flow (a diagram is strongly recommended) | 13a | For each group, the numbers of participants who were randomly assigned, received intended treatment, and were analysed for the primary outcome | 4 & Suppl. Figure 3 |
|  | 13b | For each group, losses and exclusions after randomisation, together with reasons | Suppl. Figure 3 |
| Recruitment | 14a | Dates defining the periods of recruitment and follow-up | 4 |
|  | 14b | Why the trial ended or was stopped | NA |
| Baseline data | 15 | A table showing baseline demographic and clinical characteristics for each group | Table 1 |
| Numbers analysed | 16 | For each group, number of participants (denominator) included in each analysis and whether the analysis was by original assigned groups | Tables 2, 3, supple. Tables 2-6 |
| Outcomes and estimation | 17a | For each primary and secondary outcome, results for each group, and the estimated effect size and its precision (such as 95% confidence interval) | Tables 2 & 3, supple. Tables 2-6, pg 4-6 |
|  | 17b | For binary outcomes, presentation of both absolute and relative effect sizes is recommended | 4-6 |
| Ancillary analyses | 18 | Results of any other analyses performed, including subgroup analyses and adjusted analyses, distinguishing pre-specified from exploratory | 4-6 |
| Harms | 19 | All important harms or unintended effects in each group (for specific guidance see CONSORT for harms) | 5, 6 |
| Discussion | | | |
| Limitations | 20 | Trial limitations, addressing sources of potential bias, imprecision, and, if relevant, multiplicity of analyses | 6-9 |
| Generalisability | 21 | Generalisability (external validity, applicability) of the trial findings | 6-9 |
| Interpretation | 22 | Interpretation consistent with results, balancing benefits and harms, and considering other relevant evidence | 6-9 |
| Other information | | |  |
| Registration | 23 | Registration number and name of trial registry | 10 |
| Protocol | 24 | Where the full trial protocol can be accessed, if available | 10 |
| Funding | 25 | Sources of funding and other support (such as supply of drugs), role of funders | 16 |

Citation: Schulz KF, Altman DG, Moher D, for the CONSORT Group. CONSORT 2010 Statement: updated guidelines for reporting parallel group randomised trials. BMC Medicine. 2010;8:18.
© 2010 Schulz et al. This is an Open Access article distributed under the terms of the Creative Commons Attribution License (<http://creativecommons.org/licenses/by/2.0>), which permits unrestricted use, distribution, and reproduction in any medium, provided the original work is properly cited.

*We strongly recommend reading this statement in conjunction with the CONSORT 2010 Explanation and Elaboration for important clarifications on all the items. If relevant, we also recommend reading CONSORT extensions for cluster randomised trials, non-inferiority and equivalence trials, non-pharmacological treatments, herbal interventions, and pragmatic trials. Additional extensions are forthcoming: for those and for up-to-date references relevant to this checklist, see [www.consort-statement.org](http://www.consort-statement.org)
